# Decoupling gain and feedback in coherent random lasers: experiments and simulations

**DOI:** 10.1038/srep16848

**Published:** 2015-11-18

**Authors:** Antonio Consoli, Cefe López

**Affiliations:** 1 Instituto de Ciencia de Materiales, Consejo Superior de Investigaciones Científicas, Calle Sor Juana Ines de la Cruz, 3, 28049, Madrid, Spain

## Abstract

We propose and demonstrate a coherent random laser in which the randomly distributed scattering centres are placed outside the active region. This architecture is implemented by enclosing a dye solution between two agglomerations of randomly positioned titanium dioxide nanoparticles. The same spectral signature, consisting of sharp spikes with random spectral positions, is detected emerging from both ensembles of titanium dioxide nanoparticles. We interpret this newly observed behaviour as due to the optical feedback given by back-scattered light from the scattering agglomerations, which also act as output couplers. A simple model is presented to simulate the observed behaviour, considering the amplitude and phase round trip conditions that must be satisfied to sustain lasing action. Numerical simulations reproduce the experimental reports, validating our simple model. The presented results suggest a new theoretical and experimental approach for studying the complex behavior of coherent random lasers and stimulate the realization of new devices based on the proposed architecture, with different active and scattering materials.

Random lasers (RLs) are optical devices based on disordered structures, where the feedback for lasing action is given by a random distribution of scattering particles embedded with an optically active element^1^,^2^. Opposite to conventional lasers, RLs do not present a clearly defined optical cavity as light is trapped by multiple scattering, resulting in omnidirectional emission.

Potential applications for RLs span a wide range of fields^3^. Recently, the low spatial coherence of RLs has been exploited in the first laser based speckle free imaging system^4^. In general, the ease of fabrication of such devices is an extremely attractive element that makes possible the fabrication of RLs from a wide range of materials, e.g. colloidal solutions^5^, semiconductor powders^6^, biological tissues^7^, liquid crystals^8^, optical fibres^9^ and polymer blends^10^.

The peculiar spectral signature of a coherent RL is a multimode spectrum with sharp peaks and no evident spectral periodicity^11^. The origin of such narrow spikes with sub-nanometre linewidths and placed at random frequency positions has puzzled scientists for long time^12^,^13^,^14^,^15^,^16^,^17^. From the first experimental observations^6^, it was deduced that the observed narrow linewidth was originated by coherent feedback. Consequently, this kind of emission has been referred as coherent random lasing, opposite to the incoherent random emission^5^, characterized by a single-peaked broad spectrum (few nanometres) and attributed to amplitude-only feedback.

In general, the spectrum of a coherent RL is uniquely associated to the specific random distribution of its scattering elements, which affects the spatial distribution of modes and consequently the spectrum^18^,^19^. Through optimization of the pump transverse profile, it is possible to control random lasing characteristics, e.g. excited frequencies^20^ and emission direction^21^.

All the previously reported experimental and theoretical works on coherent random lasing, consider a RL architecture based on spatially distributed feedback mechanism, in which scattering particles and active material share the same region of space. The sole exception is found in the first works on non-resonant lasing, where experiments were performed on a structure consisting of an optically active crystal and a cavity formed by one mirror and one scattering surface^22^,^23^. However, no coherent random lasing was obtained in those experiments. This kind of spectrum will be observed for the first time, only about 30 years later, by Cao and colleagues^6^ in a distributed feedback RL, consisting of randomly distributed nanoparticles of active material.

Here, we investigate the emission characteristics of a structure in which gain region and feedback elements are spatially separated. Two random agglomerations of highly scattering particles are placed near the edges of an optically pumped active medium, obtaining coherent random lasing emission.

We observe that narrow frequency modes, typical of coherent RLs, are detected at the same random frequency positions from both agglomerations of randomly distributed scattering particles. Despite the simplicity of the results, this observation poses a relevant question on the underlying physics involved: how two different disordered media contribute to generate the same spectral signature, if the spatial distribution of modes is different for each of them?

We suggest that a feedback loop is established and the entire structure is working as a single oscillator, where the active material serves as amplifying waveguide and the scattering elements act as back-scattering reflectors and output couplers.

Experimental results are presented and a simple theoretical interpretation is proposed. We based our model on the well-known round trip condition^24^, considering arbitrary frequency dependent responses of the scattering elements in amplitude and phase. An intuitive picture of the physics underlying the observed results is given and good qualitative agreement with experimental results is obtained.

## Results and Discussion

### Experimental results

Samples are obtained by deposition of high fraction aqueous suspension of TiO_2_ nano-particles on a glass substrate. After water evaporation, regions of the deposited thick layer are selectively removed, obtaining an area free of scatterers where the dye solution (Rhodamine B) is successively added. The active region is defined by illuminating a selected area of the dye using a Spatial Light Modulator (SLM) in reflection which shapes the transverse beam profile of a frequency doubled (532 nm) Q-switched Nd:YAG laser^25^. The pump energy hitting the sample can be finely tuned by changing the reflectivity of the active pixels of the SLM.

In this way we obtain a pumped area delimited by two porous “walls” of TiO_2_ with irregular and rough surfaces. Image of the sample are detected with a CCD camera and spectra are collected with a 105 μm core diameter fibre and sent to the spectrometer. Details on experimental set-up and sample fabrication are given in Methods section.

The pumped area is a rectangle with horizontal length L and vertical width W. The length L is kept smaller than the separation, *d*, between the diffusive walls of TiO_2_, so that the dye that eventually infiltrated the TiO_2_ agglomerations is not pumped.

**Figure 1:**
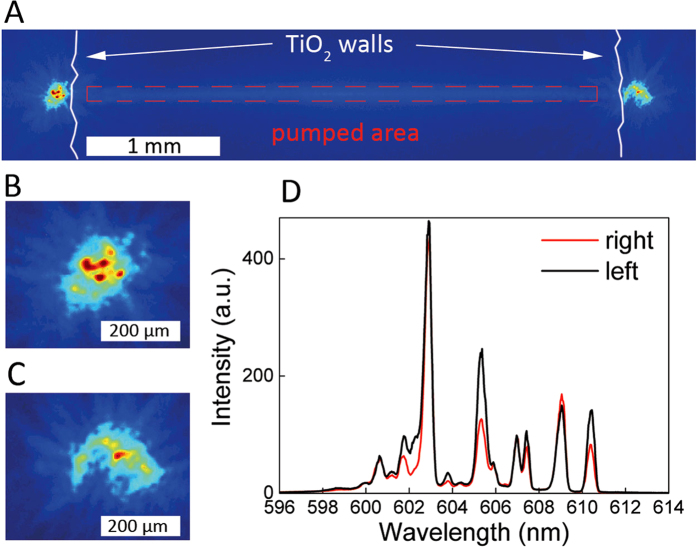
Coherent random lasing from spatially localized feedback architecture. Sample image (**A**). Zoomed view of the emitting regions in left (**B**) and right (**C**) agglomerations of TiO_2_. Measured spectra (**D**) from left (black line) and right (red line) emitter. The geometry parameters are *d* = 4.0 mm, *L* = 3.8 mm, *W* = 150 μm and the pump energy is 25 pJ/μm^2^.

In Fig. 1, we show the results obtained with *d* = 4.0 mm, *L* = 3.8 mm and *W* = 150 μm. The pump density is E_P_ = 25 pJ/μm^2^. The system is shown, in Fig. 1a, where the pumped area is the rectangular region (highlighted with red dashed line) between the diffusive walls of titanium dioxide (outlined with white lines). The regions of the TiO_2_ agglomerations facing the two horizontal ends of the pumped area emit intense light in the out of the plane direction: a zoomed view is shown in Fig. 1b,c, for left and right TiO_2_ region, respectively. Local maxima and minima of intensity suggest that spatial interference due to multiple scattering is taking place in each region. Although similar “hot spots” are common in RL with distributed feedback, we recall that in our device no gain is present inside the clusters, posing a relevant difference with conventional RLs.

The spectra collected from each TiO_2_ agglomerations are shown in Fig. 1d. Typical signature of coherent random lasing is observed: a multi-mode emission with narrow linewidths at random frequencies. But most importantly, the same frequency peaks are detected from both scattering elements.

Experiments performed with many different samples show the same behavior: one spectral signature consisting of narrow linewidths at random frequencies is detected from both TiO_2_ agglomerations.

Commonly, a RL is associated to a unique spectrum which depends on the specific random distribution of scattering particles of the considered device. As an example, this concept has been proposed for potential application in optical tagging based on RLs^3^. However, in our structure, a different mechanism for lasing action must be considered, as two different random distributions of scattering particles contribute to the same spectral signature. Small differences in intensity peaks are attributed to the direction dependent output coupling efficiency of each TiO_2_ element.

We propose a simple interpretation of the observed results. The spontaneous emission from the pumped area is amplified along L due to the inverted population along this direction. The amplified spontaneous emission (ASE) exits the pumped area and reaches the TiO_2_ diffusive agglomerations after being poorly attenuated in the unpumped dye between the active area and the TiO_2_ agglomerations. The back-scattered light re-enters the active region, closing an optical feedback loop and provoking lasing action. The two random media act as a pair of frequency- and direction- selective reflectors and the rectangular pumped area as a bidirectional amplifying waveguide.

**Figure 2:**
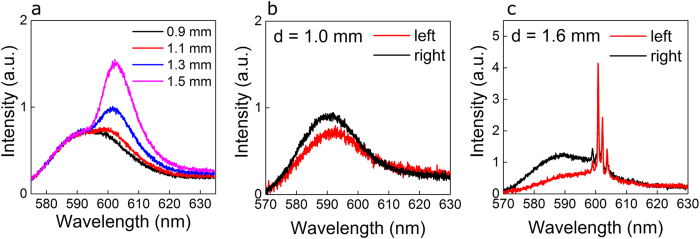
Below and above threshold emission regimes. Spontaneous emission and ASE detected from the dye solution free of scatterers, obtained with different pump lengths between 0.9 mm and 1.5 mm (**a**). Spectra collected from left (red lines) and right (black lines) diffusive mirrors in different laser structures with d = 1.0 mm (**b**) and d = 1.6 mm (**c**) and with L = 0.9·d.

The ASE from the dye solution has been characterized by illuminating a stripe of the dye solution with no scatterers and collecting the light deflected from the edges of the dye drop (more details in Supplementary Information material). The length of the stripe is varied between 0.9 mm and 1.5 mm and the measured spectra are shown in Fig. 2a. A broad spectrum with peak intensity at 590 nm is detected for small values of the pump length L. As L is increased, the emission peak shifts at 600 nm and the spectral width narrows to about 10 nm. The peak spectral shift is attributed to the partial overlap of absorption and fluorescence bands in Rhodamine dyes^26^. The peak intensity increase is due to the longer amplification path along which the ASE is travelling as L is increased.

The lasing threshold of the proposed laser architecture is the result of the gain contribution given by the rectangular pumped area and the losses introduced by the TiO_2_ agglomerations. At constant pump energy density, longer devices lase, while shorter devices only emit below threshold with ASE.

This is shown in Fig. 2b,c, where two different device geometries are considered with two values of d, with L kept slightly shorter than d and pumped with the same energy density E_P_ = 25 pJ/μm^2^. For d = 1.0 mm, a broad noisy spectrum with FWHM of about 18 nm is detected from both laser ends. In this condition, the ASE from the pumped area is scattered off the plane from the TiO_2_ regions, and no lasing action occurs. For d = 1.6 mm, lasing takes place and the same set of modes is observed from both ends of the laser, superimposed on the broad ASE background. With this geometry and at the same energy density E_P_, the device is lasing slightly above threshold.

**Figure 3:**
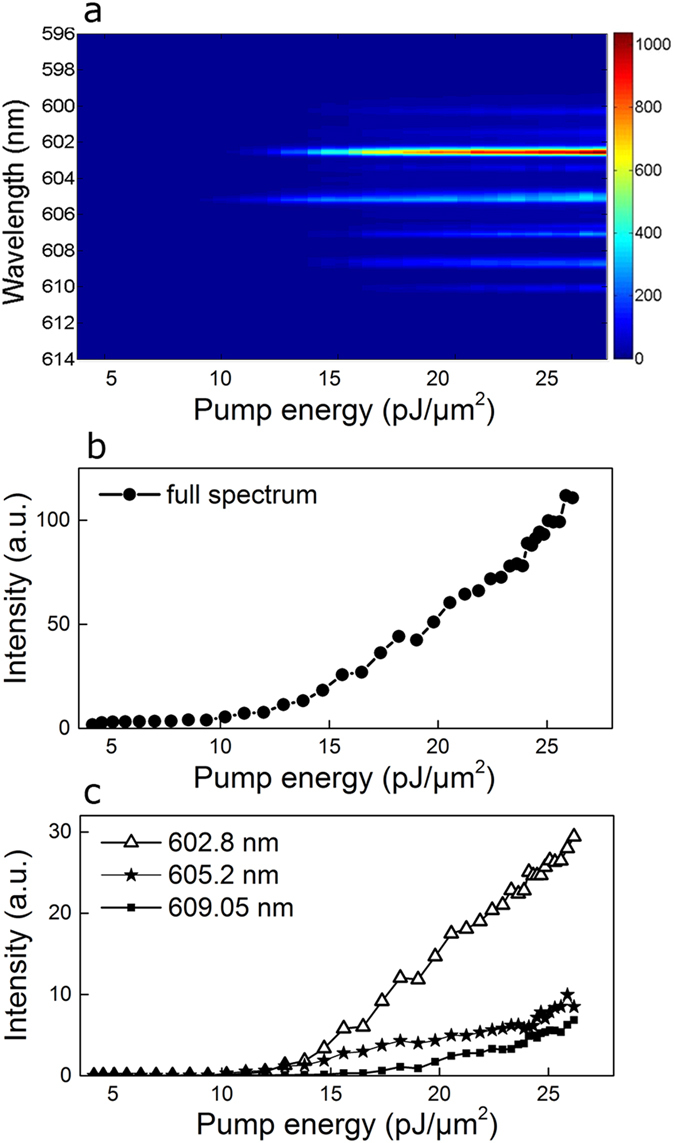
Pump dependent emission spectrum. Measured spectra as a function of the pump energy (**a**). Total detected intensity integrated over the frequency range of measurement (**b**). Peak intensity of three modes at 602.8 nm (triangles), 605.2 nm (star symbol) and 609.05 nm (full squares), as a function of the pump energy (**c**).

Using the device geometry shown in Fig. 1, we measured the emitted spectra from one laser end as a function of E_P_. Results are shown in Fig. 3a. For low values of E_P_, only a broad ASE spectrum is measured. As the pump energy is increased, two modes start to lase at 602.8 nm and 605.2 nm, for E_P_ = 13 pJ/μm^2^. At higher energies, more peaks appear and grow with increasing pump energy. Notably, the spectral position of the excited modes is constant with E_P_.

In Fig. 3b, the spectral intensity integrated over the entire frequency range of detection is plotted. A clear threshold knee is observed between E_P_ = 10 pJ/μm^2^ and E_P_ = 15 pJ/μm^2^ and an almost linear increase is observed for higher values of E_P_.

The first two lasing mode and a further mode at 609.5 nm are selected for monitoring their growth as a function of the pump energy. Figure 3c shows their intensities as a function of the pump energy. The same threshold (E_P_ = 13 pJ/μm^2^) is observed for modes lasing at 602.8 nm and 605.2 nm, after which they grow with different slopes. The mode at 609.05 nm has a higher threshold of 17 pJ/μm^2^ and then it grows with moderate slope value.

Qualitatively, the same behaviour has been observed in experiments performed with other samples. The following common features have been always detected: narrow spikes at randomly distributed frequencies which vary with the considered sample, same spectrum from both laser ends, constant spectral positions of lasing modes with pump energy and different thresholds and slopes for different modes.

Additionally, we observed that, if the width W is increased, more modes are excited, leading to a broadened spectral profile^5^, in which strong non-linear interactions between modes can take place, as in mode-locked operation^27^. In the present manuscript, however, we limit our experimental work and theoretical analysis to the case of a linear device with few, non-interacting modes, for sake of simplicity.

### Theoretical model and numerical simulations

In order to understand the observed lasing regime, we first consider the nature of backscattered radiation from a rough surface. It is well known that the backscattered light intensity from diffusive reflectors is maximum for θ = 0, where θ is the angle between the incident and backscattered light directions^28^. This is due to the coherent contribution for θ = 0, where the scattered light has a 2π phase delay with respect to the incident light. The backscattering cone is the resulting intensity distribution as a function of the detection angle, being maximum at θ = 0 and decreasing for larger θ. We believe we can safely assume that our quasi two dimensional structures obey this behaviour.

In our structure, both TiO_2_ walls act as backscattering surfaces. The phase difference between the incident and back-scattered light is exactly 2π for θ = 0. However, considering that the width of the pumped area is not infinitely narrow, back-scattered light from one TiO_2_ region propagates and is amplified along directions with back-scattering angles different from zero. Along these paths the phase contribution of the diffusive reflectors of TiO_2_ assumes a random value, as belonging to the incoherent component of the back-scattering cone, being θ ≠ 0.

**Figure 4:**
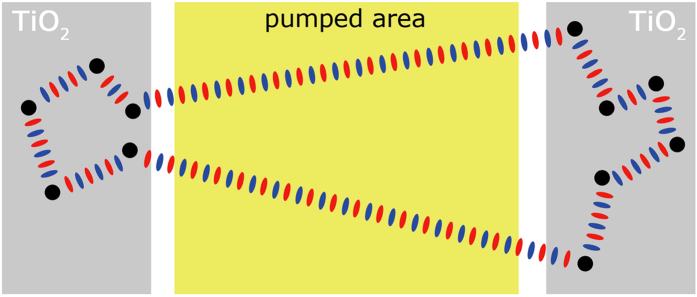
Schematic description of the round trip phase condition. The device architecture is schematically depicted with pumped region and TiO_2_ elements given by yellow and gray areas, respectively. Scattering centers are shown as black full circles and travelling light is pictured with intercalated red and blue ellipses, schematically rendering the wavefronts.

We sketch this concept with a schematic illustration in Fig. 4, showing the case of a single lasing frequency mode. Schematically, the scattering path in each TiO_2_ element (grey areas) add an arbitrary phase contribution to the back-scattered light, which travels back to the other laser end, where another arbitrary phase contribution is added before the light is back-scattered again into the active region (yellow area). If the considered frequency adds up to an integer number of wavelength periods in a round trip, given the arbitrary phase contribution of each diffusive reflector, it will be allowed in the cavity and grow.

Contrary to the case of a ring laser, a closed trajectory allows only one frequency mode, the one that satisfies the selection criteria given by the phase contributions of the diffusive reflectors in one round trip.

Concerning the amplitude of the back-scattered wave at one frequency, it depends on the scattering losses of the specific path along which that frequency is traveling. This equals to say that losses of the TiO_2_ regions vary with frequency.

The previous considerations can be modelled in a simple way, considering that both the amplitude and phase response of the back-scattering TiO_2_ elements are frequency dependent and have arbitrary spectral profiles. Let us express the complex spectral response of the back-scattering reflectors as R_1,2_(*v*)·exp[*i*ϕ_1,2_(*v*)], where R_1,2_(*v*) are the amplitude components, ϕ_1,2_(*v*) the phase components, *v* the frequency variable and *i* the imaginary unit.

We introduce R_1,2_(*v*)·exp[iϕ_1,2_(*v*)] into the well-known round trip condition of a conventional laser^23^ and obtain:


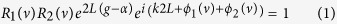


*L* being the active medium length, *g* the linear gain, *α* the absorption and *k* the wave vector.

The main point of the proposed model is that the spatial complexity of the problem is translated into the frequency domain. In this vision, the path lengths travelled inside the TiO_2_ agglomerations are modelled with arbitrary frequency dependent responses, where the losses are taken into account in the amplitude part and the coherent contribution in the phase part. A similar argument allows us to neglect the spatial variation of L, given by L ± ΔL, where ΔL is a random distribution of lengths in the order of tens of micrometres, due to the roughness of the TiO_2_ agglomerations. In fact, the gain and losses contributions into the active medium, are poorly affected by ΔL as ΔL ≪ L and the phase delay introduced only adds a random phase, that can be considered as already included in the term ϕ_1_(*v*) + ϕ_2_(*v*).

In case a large number of modes (e.g. >10) is obtained, as it has been observed with increased width W, non-linear effects, such as strong mode interactions^27^ should be also considered. In this manuscript we limit our analysis to the linear region of operation of the device.

Equation 1 can be separated into an amplitude condition:


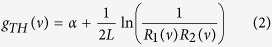


where g_TH_(*v*) is the frequency dependent threshold gain, and into a phase condition:


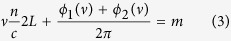


where *m* is an integer number and the relation k = 2π*v*n/c has been used, with *n* being the refractive index and *c* the speed of light in vacuum.

The amplitude equation (Eq. 2) simply states that for reaching threshold, the gain must be equal to the sum of a fixed term of internal losses and a frequency dependent term, given by the joint contribution of the reflective amplitude components of the diffusive reflectors. In a classical laser, the mirror losses are constant with frequency and consequently losses are the same for all wavelengths.

The phase equation (Eq. 3) would reduce to the classical Fabry-Perot solution with equally spaced, c/(2 nL), frequency modes if the phase term (ϕ_1_(*v*) + ϕ_2_(*v*))/2π is constant, as it is the case with specular mirrors. Considering our system, satisfaction of Eq. 3 represents the lucky event in which, given a certain frequency *v* and the phase contribution (ϕ_1_(*v*) + ϕ_2_(*v*))/2π, an integer number of wavelength periods fits the round trip.

**Figure 5:**
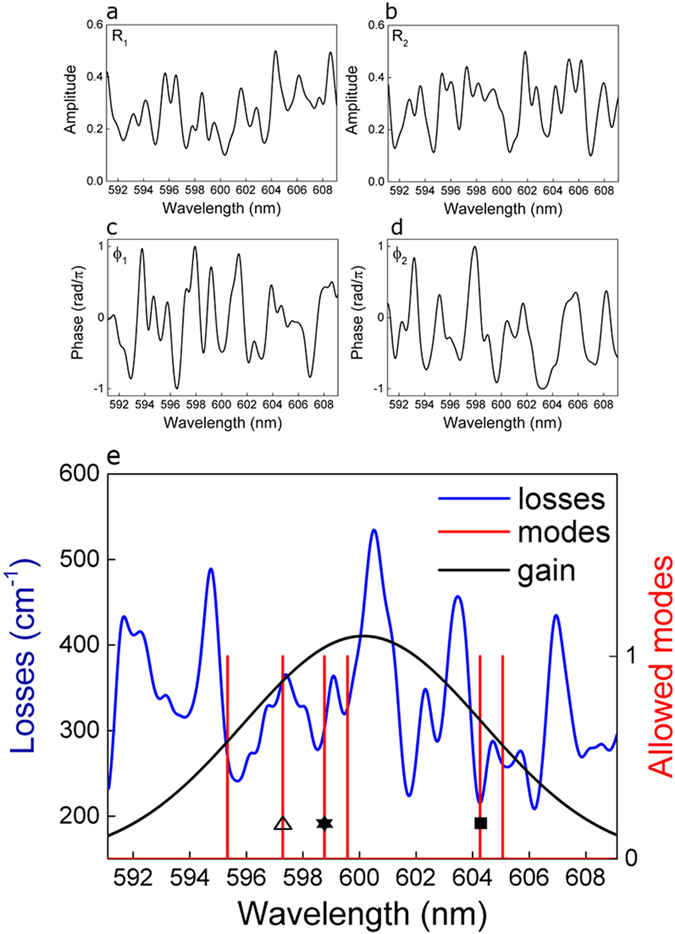
Simulation results: gain, losses and allowed modes. Amplitude functions R_1_ (**a**) and R_2_ (**b**) versus wavelength. Phase responses ϕ_1_ (**c**) and ϕ_2_ (**d**) versus wavelength. (**e**) Losses (blue line) and allowed modes (red line) after evaluation of Eq. 2 and Eq. 3. The gain shape (black line) is also shown. Three modes at 597.3 nm, 598.8 nm and 604.3 nm are highlighted with symbols: triangles, stars and full square, respectively.

Reflectivities R_1_(*v*) and R_2_(*v*) are numerically implemented with arbitrary shapes, see Fig. 5a,b, respectively. We consider smooth profiles with few peaks contained in the gain spectral window with arbitrarily minimum and maximum reflectivity values. Qualitatively similar results are obtained with different parameters: sharper peaks and larger reflectivity excursion result in a spread distribution of modal threshold values.

Phase responses ϕ_1_(*v*) and ϕ_2_(*v*) are numerically implemented with smooth spectral profiles and arbitrary values between −π and + π, with a resolution of 0.1 mrad, as shown in Fig. 5c,d, respectively.

Amplitude and phase spectral profiles are constructed from the sum of gaussian curves which are randomly distributed in the spectral range of interest. Once the profile is obtained, it is rescaled imposing maximum and minimum values. Sharper or smoother profiles are obtained by varying two parameters: the number of curves in a fixed wavelength range and its FWHM. In the simulations results presented in Fig. 5, we impose a FWHM of 0.6 nm and a number of 40 curves in 10 nm. As the Gaussian profiles are free to overlap randomly, the full profiles obtained at each simulation vary strongly, maintaining however smooth, sub-nanometer spectral features. Smoother spectral shapes are obtained increasing the FWHM and decreasing the number of Gaussian curves. As an example, in Supplementary Information material we present results obtained with smoother profiles, which have been constructed with 5 gaussian curve in a wavelength range of 10 nm and with FWHM = 1.8 nm.

The key element in the numerical construction of R_1,2_(*v*) and ϕ_1,2_(*v*) is that their values have to be spectrally uncorrelated, *i.e.* varying arbitrarily from frequency to frequency. We have numerically implemented different kind of spectral profiles, from very abrupt spectral changes (presented in Supplementary Information material) to smooth transitions, as the ones shown in Fig. 5. Qualitatively, the same results are obtained in both cases.

Other simulations parameters are set according to experimental conditions, with length L = 4 mm, dye refractive index n = 1.5 and internal losses deduced from dye characterization, obtaining α = 9.8 cm^–1^. The gain spectral profile is assumed gaussian, centered at 600 nm and with a FWHM of 10 nm. The frequency vector spans 15 THz (18 nm at 600 nm) with a resolution of 150 MHz (0.18 pm at 600 nm). Equations 2 and 3 are implemented in the frequency domain and results are plotted as a function of wavelength, in order to easily compare with experimental results.

In numerical simulations, we first construct R_1,2_(*v*) and ϕ_1,2_(*v*) and then use R_1,2_(*v*) in Eq. 2, for obtaining the losses profile that give the lasing thresholds, and ϕ_1,2_(*v*) in Eq. 3, for obtaining the allowed frequency modes.

In Fig. 5e, we plot the calculated losses, corresponding to the right side of Eq. 2, the allowed modes, resulting from evaluation of Eq. 3, and the gain shape. Few allowed frequencies are observed (six modes in the presented simulation), with irregular spacing and no spectral periodicity. Depending on their spectral position, each mode sees a different balance between gain and losses, resulting in different thresholds and slopes.

The number of allowed modes and their positions depend on how many times and where, Eq. 3 is satisfied. These are unpredictable events, given the arbitrary and spectrally uncorrelated contributions of ϕ_1_(*v*) and ϕ_2_(*v*) at each frequency, resulting in a random number of randomly distributed frequency modes.

If the random phases are changed with respect to the presented simulation results, different sets of modes are obtained, which correspond to different pairs of diffusive mirrors.

**Figure 6:**
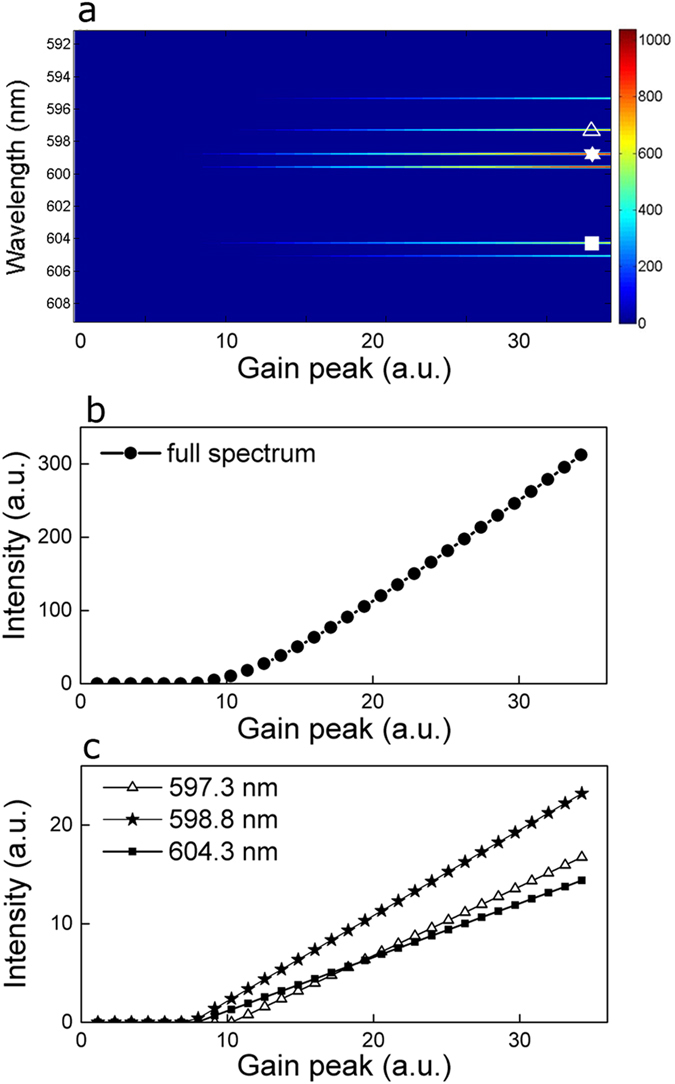
Simulation results with increasing gain. (**a**) Emission spectra as a function of gain, with three modes at 597.3 nm, 598.8 nm and 604.3 nm, highlighted with symbols: triangles, stars and full squares, respectively. (**b**) Spectral intensity integrated over the full frequency range as a function of gain. (**c**) Peak intensity of three modes at 597.3 nm (triangles), 598.8 nm (stars) and 604.3 nm (full squares).

In simulation, the spectral gain is increased and Eq. 2 is evaluated, given the allowed frequency modes from Eq. 3, obtaining the emission spectrum as a function of the gain. Specifically, the gain curve is multiplied by an increasing factor, maintaining the gain FWHM and central wavelength. Results are shown in Fig. 6a. All modes have different threshold values and different slopes.

The spectral energy integrated over the full frequency range is plotted in Fig. 6b. A linear increase is observed after the first lasing modes appear. A detailed view of the mode evolution is given in Fig. 6c, where the intensity peaks of three modes at 597.3 nm, 598.8 nm and 604.3 nm are plotted as a function of the gain. Modes at 598.8 nm and 604.3 nm present the same threshold, despite the fact that at 598.8 nm more gain is available: this is because of the low losses at 604.3 nm (see Fig. 5e). The net gain must be evaluated at each frequency and low gain spectral region where a frequency mode is available can lase first if losses are small enough.

As opposite example, the frequency mode at 597.3 nm has higher threshold despite experiencing near peak gain, due to the high scattering losses at its frequency.

## Conclusions

In conventional RLs, the feedback for lasing action is given by the ensemble of randomly distributed scattering centers embedded with the active material. The feedback mechanism is then spatially distributed along the laser structure. Here, we report coherent random lasing from a novel architecture in which the feedback elements are spatially localized at the edge of an amplifying active region.

We use two random agglomerations of highly scattering nanoparticles which back-scatter the received radiation into the active material and close a feedback loop. Multi-mode, narrow linewidth emission, typical of coherent random lasing, is observed from both scattering elements, as they are also acting as output couplers, similarly to conventional mirror-based lasers.

By establishing a simple but effective analogy with the round trip condition of standard lasers, we were able to construct a simple model for intuitive understanding of the observed behavior. Our model is based on the assumption that frequency dependent amplitude and phase response of the back-scattering surfaces have arbitrary spectral profiles.

From a practical point of view, the proposed architecture is appealing for its flexibility, as it can be realized with a rich variety of active elements, e.g. semiconductor, fibre or crystals, and scattering elements, provided that the active medium is enclosed between two back-scattering rough surfaces. From a fundamental physics point of view, the reported results are interesting as they bring a new perspective on coherent random lasing, due to the first experimental observation of a lasing feedback loop between two back-scattering surfaces. The model we propose gives an intuitive and simple explanation of the observed results and helps the understanding of random lasing action, by considering the amplitude and phase contributions of the scattering regions as feedback elements for lasing.

## Methods

### Sample fabrication

**Figure 7:**
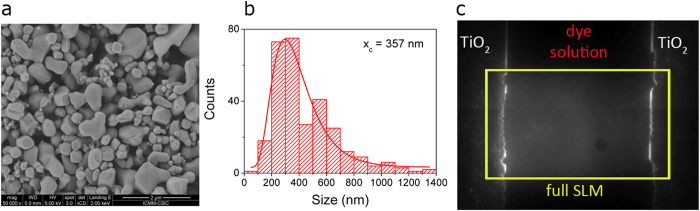
Sample characterization. (**a**) SEM image of the TiO_2_ powder used in sample preparation. (**b**) Particle size distribution extracted from SEM images. (**c**) Image of a region of a sample, consisting of dye solution (dark area) and TiO_2_ agglomerations (reddish areas). The full SLM screen (see next section) is highlighted with a yellow rectangle.

The titanium dioxide powder (rutile, Sigma Aldrich 224227) used in sample preparation is first characterized by a Scanning Electron Microscope (SEM) analysis. A SEM image is shown in Fig. 7a. Irregular shapes and very different particle size are observed. We analysed a set of ten images obtained from our SEM measurements and extracted a mean particle size of 357 nm, as shown in Fig. 7b.

A solution is prepared with 5 mL of distilled water and 20 mg of TiO_2_ powder. A small quantity (20 μL) of the solution is then dropped onto a glass substrate, which has been previously hydrophilized with a 20 min bath in chromic acid. The substrate is then placed below a 60 W lamp for 20 minutes, after this time all water is evaporated and a layer of TiO_2_ is fixed to the substrate.

A selected region of the TiO_2_ layer is mechanically removed, obtaining an area of the sample free of TiO_2_ and delimited by two rough regions of TiO_2_. A solution of Rhodamine B (0.5 vol. % in equal parts of ethanol and ethylene glycol) is dropped (10 μL) onto the substrate. Two 50 μm thick aluminium spacers are placed outside the dye drop, and a plastic cover slip is then positioned onto the sample. Our sample is now squeezed between the glass substrate and the plastic coverslip and it consists of two large “walls” of TiO_2_ placed at the edges of the dye solution. A typical image is shown in Fig. 7c, where also the full screen of the Spatial Light Modulator, SLM, (see next section) is shown.[Fig f8]

### Experimental set-up

**Figure 8 f8:**
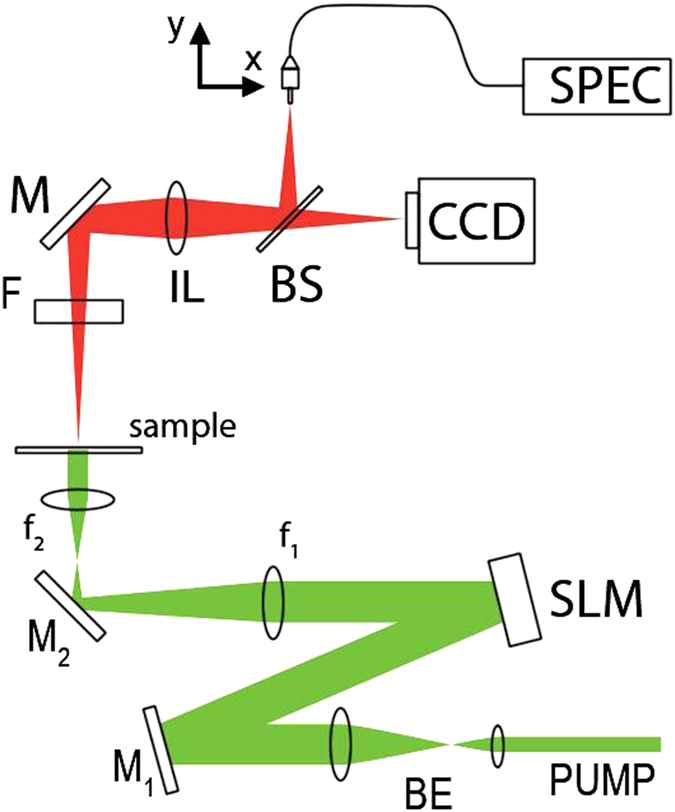
Experimental set-up. Details are given in the text.

The experimental set-up is shown in Fig. 8. The pump source is a Q-switched Nd:YAG laser, frequency doubled at 532 nm (Litron NanoT250), emitting 10 ns pulses, at 10 Hz repetition rate. The pump transverse profile is shaped with a Spatial Light Modulator (SLM, Holoeye LCR-1080) operating in amplitude^24^. The SLM liquid crystals screen is computer controlled and an arbitrary mask is sent to the device in the form of a matrix (1920 × 1200 elements) of black (non-reflective) and white (reflective) pixels. The amount of the reflected light is controlled by imposing a variable gray level to the active pixels of the SLM.

The SLM image is reduced (×0.15) with a pair of convex lenses with focal length f1 = 20 cm and f2 = 3 cm. Polarizing dichroic mirrors (M1, M2) are used in order to have linearly polarized light hitting the sample and a beam expander (BE, ×5) is used to uniformly shine the SLM.

An optical filter (F) is placed after the sample for stopping the pump light. A large area of the sample (5.7 mm × 4.27 mm) is imaged (1:1) onto a CCD (Pixelink model PL-B776F) camera and on a connectorized fibre end (105 μm core diameter) with an imaging lens (IL, 15 cm focal length) and a beam splitter (BS). The fibre core acts as a spatial filter which collects a circular area on the sample with the dimension of its core. Fibre collected light is sent to a 303-mm focal length spectrograph (SPEC, Andor, Shamrock 303) connected to a low-noise charge-coupled device array (Andor, iDus Spectroscopy CCD). Light is collected from different regions of the sample by moving the fibre horizontally and vertically with two computer controlled translation stages.

## Additional Information

**How to cite this article**: Consoli, A. and López, C. Decoupling gain and feedback in coherent random lasers: experiments and simulations. *Sci. Rep.* **5**, 16848; doi: 10.1038/srep16848 (2015).

## Supplementary Material

Supplementary Information
