# Supplementary material for: Decoupling gain and feedback in coherent random lasers: experiments and simulations
Source: Sci Rep. 2015 Nov 18;5:16848. doi: 10.1038/srep16848 (PMC4649543; doi:10.1038/srep16848)
Supplement: Supplementary Information [file srep16848-s1.pdf]

Decoupling gain and feedback in coherent random lasers: experiments and simulations

Antonio Consoli[1]* and Cefe López[1]

[1]Instituto de Ciencia de Materiales de Madrid, Consejo Superior de Investigaciones Científicas, Calle Sor Juana Ines de la Cruz 3, 28049 Madrid, Spain

*antonio.consoli@cisc.es

Supplementary information

ASE characterization.

We characterized the ASE flux by illuminating a stripe of width W = 50 µm and variable length L in a region where only the dye solution is present and no clusters are observed. One end of the stripe is placed at the edge of the dye solution with air and the portion of light reflected towards our detection system is collected at the spectrograph, see Fig. SI1a. We varied the stripe length L, between 900 µm and 4.0 mm, and measured the collected spectrum at each value of L, as shown in Fig. SI1b. Measured spectra are integrated over the entire frequency range of the measurement and results are plotted with full dots in Fig. SI2a.

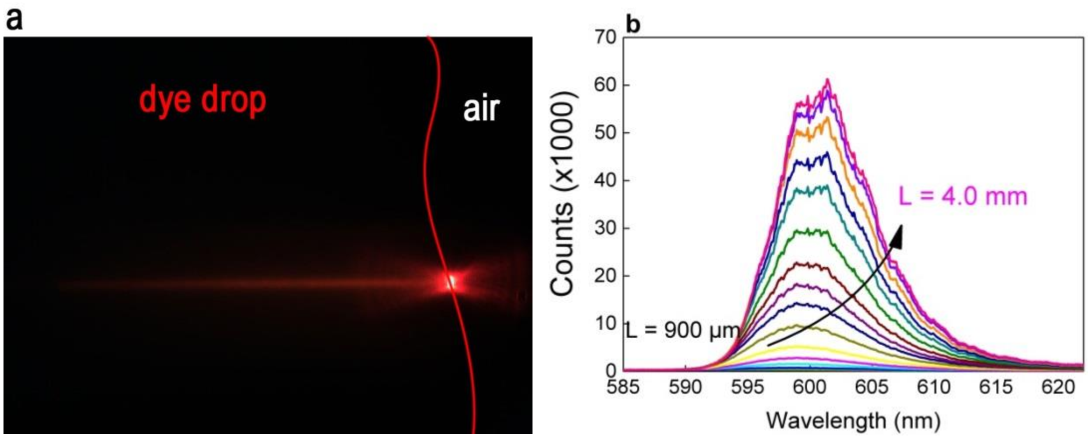

Fig. SI1. ASE characterization. (a) Image of the sample where measurements for ASE characterization are performed, a red line is superposed to the image in order to highlight the edge between the dye drop and air. The pumped stripe and the reflected light at the edge are visible. (b) Measured spectra for L between 900 µm and 4.0 mm, with steps of 200 µm.

In order to estimate the optical gain per unit length, we perform a least square fit to the measured intensity values, using the equation:

$$I(z) = A_0 \cdot g^{-1} \cdot \exp[(g \cdot z) - 1] \qquad \text{Eq. S1}$$

where I(z) is the ASE intensity as a function of the stripe length z, A_0 is a scaling factor and g the modal gain as defined in [1]. Parameters are fitted to measured data, obtaining g = 11.2 ± 1.1 cm^{-1}. The fitting procedure is evaluated by performing a chi-squared test (χ^2 = 0.07), giving a goodness of fit within a significance level of 5%.

We also estimated the absorption of the non-pumped dye with a similar experiment. A stripe with fixed length L = 900 μm is illuminated and the distance, L_{dist} , between one stripe end and the dye/air edge is varied by moving the sample. Spectra are measured for each value of L_{dist} by moving accordingly the fiber collector, for $0 < L_{dist} < 1.75$ mm. Collected spectra are integrated over the frequency range and normalized to the intensity obtained for $L_{dist} = 0$. Results are plotted in Fig. SI2b, with full dots. We performed an exponential fit to $\exp(-\alpha \cdot x)$ and obtained $\alpha = 9.8$ cm^{-1}.

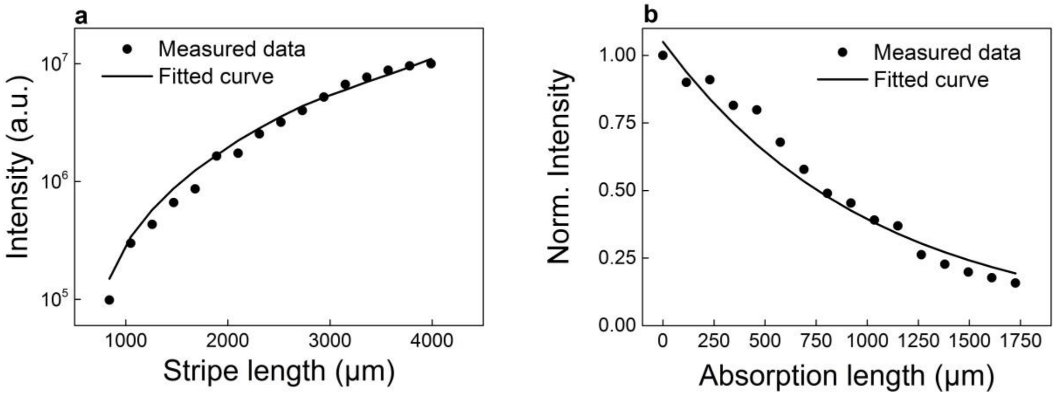

Fig. SI2. Gain and absorption coefficient of the dye. (a) Integrated measured intensity obtained increasing L, from 900 μm to 4.0 mm: measured data (full dots) and fitted curve (black line). (b) Integrated measured intensity obtained increasing L_{dist}, from 0 to 1.75 mm: measured data (full dots) and exponential fit (black line).

Additional simulation results.

In this section we present the results obtained in simulations with different spectral profiles of $R_{1,2}(\nu)$ and $\phi_{1,2}(\nu)$.

In a first set of simulations, $R_{1,2}(\nu)$ and $\phi_{1,2}(\nu)$ are constructed with uniform random distributions of values, with abrupt changes between adjacent frequencies.

In Fig. SI3a, R_1 is shown and a zoomed view is given in Fig. SI3b. The phase profile ϕ_1 is shown in Fig. SI3c, and a zoomed view in Fig. SI3d. R_2 and ϕ_2 are not shown for sake of simplicity.

In Fig. SI3e, the losses, gain curve and allowed modes are shown. As in the case of smoother profiles, reported in the manuscript, modes are randomly distributed where Eq. 3 is satisfied. Losses are noisy, reflecting the randomness imposed to R_1 and R_2. An enlarged view (on a 0.02 nm wavelength range) of the losses, gain curve and allowed modes is shown in Fig. SI3f.

The corresponding emission spectra and the total intensity as a function of the gain peak are plotted in Fig. SI4a and SI4b, respectively. Three modes of the spectrum are selected at 599.4 nm, 601.3 nm and 606 nm and their peak intensities are plotted in Fig. SI4c as a function of the peak gain.

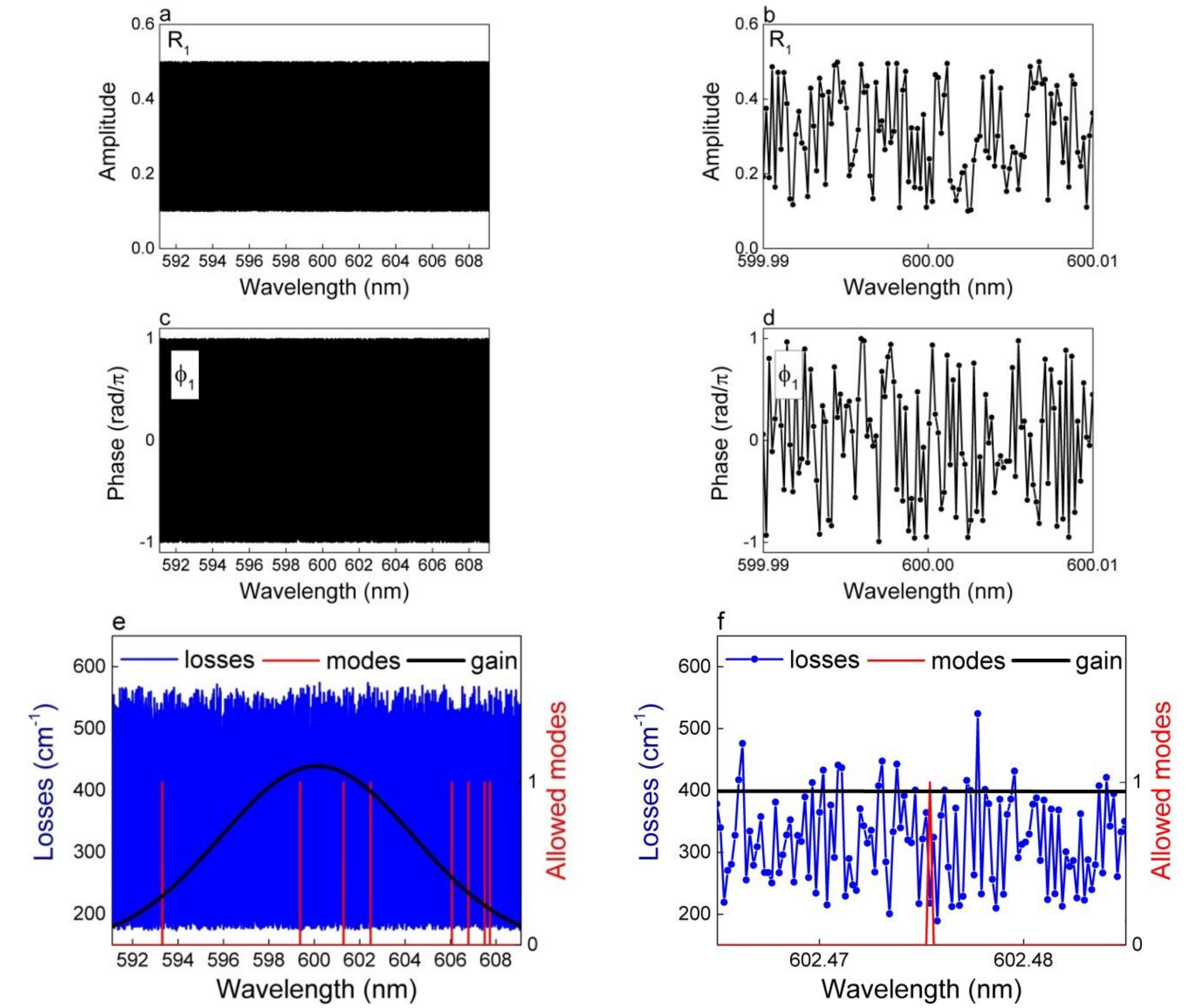

Fig. SI3. Simulations results with abrupt profiles of $R_{1,2}$ and $\phi_{1,2}$. Spectral profile of R_1 (a), with a zoomed view in (b), and of ϕ_1 (c) with a zoomed view in (d). (e) The gain curve (black line), allowed modes (red line) and losses (blue line) with zoomed view in (f).

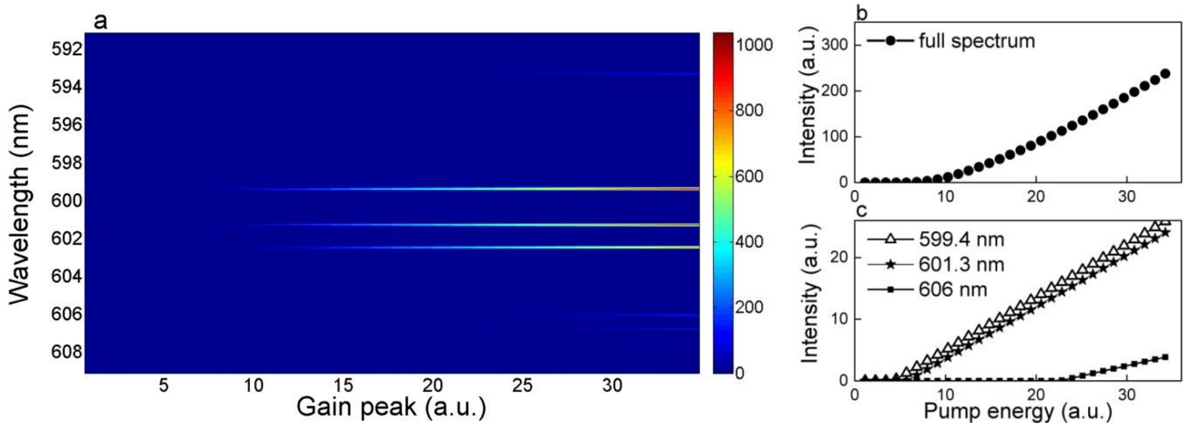

Fig. SI4. Simulated emission spectra as a function of the gain peak for abrupt spectral profiles of $R_{1,2}$ and $\phi_{1,2}$. (a) Emission spectra as a function of the gain peak. (b) Total intensity. (c) Peak intensity of three modes at 599.4 nm (triangles), 601.3 nm (star symbols) and 606 nm (squares)

In a second set of simulations, $R_{1,2}(\nu)$ and $\phi_{1,2}(\nu)$ are constructed with smooth profiles. Amplitude and phase spectral profiles are constructed from the sum of gaussian curves which are randomly distributed in the spectral range of interest. Few random peaks and smooth transitions are obtained with 5 gaussian curve randomly distributed in a wavelength range of 10 nm and with FWHM = 1.8 nm. Spectral profiles of R_1, R_2, ϕ_1 and ϕ_2 are shown in Fig. SI5a, SI5b, SI5c and SI5d, respectively.

In Fig. SI5e, the losses, gain curve and allowed modes are shown. Also here, modes are randomly distributed where Eq. 3 is satisfied. Losses have a smooth profile, reflecting the spectral shapes of $R_1 \cdot R_2$.

The corresponding emission spectra and the total intensity as a function of the gain peak are plotted in Fig. SI6a and SI6b, respectively. Three modes of the spectrum are selected at 595.7 nm, 597.6 nm and 601.9 nm and their peak intensities are plotted in Fig. SI6c as a function of the peak gain.

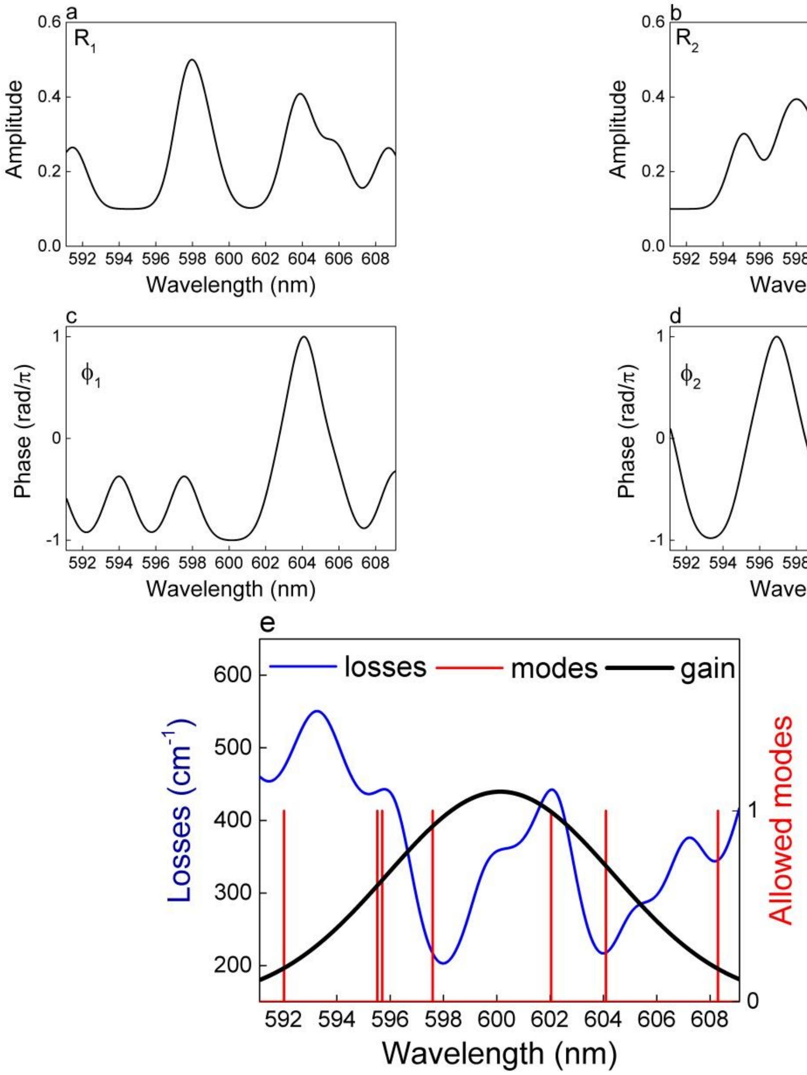

Fig. SI5. Simulations results with smooth profiles of $R_{1,2}$ and $\phi_{1,2}$. Spectral profile of R_1 (a), R_2 (b), ϕ_1 (c) and ϕ_2 (d). (e) The gain curve (black line), allowed modes (red line) and losses (blue line).

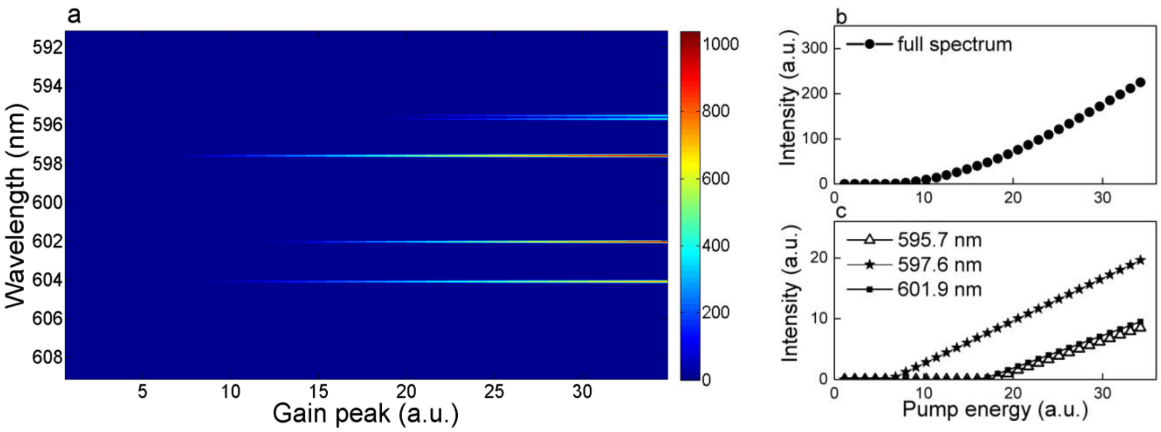

Fig. SI6. Simulated emission spectra as a function of the gain peak for smooth spectral profiles of $R_{1,2}$ and $\phi_{1,2}$. (a) Emission spectra as a function of the gain peak. (b) Total intensity. (c) Peak intensity of three modes at 595.7 nm (triangles), 597.6 nm (star symbols) and 601.9 nm (squares)

References

[1] Dal Negro, L., Bettotti, P., Cazzanelli, M., Pacifici, D. & Pavesi, L. Applicability conditions and experimental analysis of the variable stripe length method for gain measurements. *Opt. Commun.* **229**, 337-348 (2004)
